# Fiber Optic Sensors for Temperature Monitoring during Thermal Treatments: An Overview

**DOI:** 10.3390/s16071144

**Published:** 2016-07-22

**Authors:** Emiliano Schena, Daniele Tosi, Paola Saccomandi, Elfed Lewis, Taesung Kim

**Affiliations:** 1 Universita’ Campus Bio-Medico di Roma, Unit of Measurements and Biomedical Instrumentation, via Alvaro del Portillo 21, 00128 Roma, Italy; e.schena@unicampus.it; 2 School of Engineering, Nazarbayev University, 53 Kabanbay Batyr, 01000 Astana, Kazakhstan; daniele.tosi@nu.edu.kz; 3 Institute of Image-Guided Surgery (IHU), S/c Ircad, 1 place de l’Hôpital, 67091 Strasbourg Cedex, France; paola.saccomandi@ihu-strasbourg.eu; 4 Optical Fibre Sensors Research Centre (OFSRC), University of Limerick, V94 T9PX Limerick, Ireland; elfed.lewis@ul.ie; 5 School of Mechanical Engineering & SAINT, Sungkyunkwan University, 53 Myeongnyun-dong 3-ga, Jongno-gu, 110-745 Suwon, Korea; tkim@skku.edu

**Keywords:** fiber optic sensors, temperature monitoring, medical applications, minimally invasive thermal treatments

## Abstract

During recent decades, minimally invasive thermal treatments (i.e., Radiofrequency ablation, Laser ablation, Microwave ablation, High Intensity Focused Ultrasound ablation, and Cryo-ablation) have gained widespread recognition in the field of tumor removal. These techniques induce a localized temperature increase or decrease to remove the tumor while the surrounding healthy tissue remains intact. An accurate measurement of tissue temperature may be particularly beneficial to improve treatment outcomes, because it can be used as a clear end-point to achieve complete tumor ablation and minimize recurrence. Among the several thermometric techniques used in this field, fiber optic sensors (FOSs) have several attractive features: high flexibility and small size of both sensor and cabling, allowing insertion of FOSs within deep-seated tissue; metrological characteristics, such as accuracy (better than 1 °C), sensitivity (e.g., 10 pm·°C^−1^ for Fiber Bragg Gratings), and frequency response (hundreds of kHz), are adequate for this application; immunity to electromagnetic interference allows the use of FOSs during Magnetic Resonance- or Computed Tomography-guided thermal procedures. In this review the current status of the most used FOSs for temperature monitoring during thermal procedure (e.g., fiber Bragg Grating sensors; fluoroptic sensors) is presented, with emphasis placed on their working principles and metrological characteristics. The essential physics of the common ablation techniques are included to explain the advantages of using FOSs during these procedures.

## 1. Introduction

Minimally invasive techniques have gained widespread recognition for tumor treatment as an alternative to traditional surgery and to treat patients who are not candidates for surgery [1]. A particular family of minimally invasive techniques is represented by thermal ablation procedures, which induce either a localized temperature increment (Laser Ablation (LA), Radiofrequency Ablation (RFA), High Intensity Focused Ultrasound (HIFU), and Microwave Ablation (MWA)) or decrement (cryoablation) to kill the whole tumor while sparing the surrounding healthy tissue. Their main advantages over traditional surgery are primarily related to the possibility of performing the ablation procedures through percutaneous, endoscopic, or extracorporeal guidance, hence minimizing the physical trauma to the patient, avoiding adverse complications, reducing the demand for general anesthesia, and treating inoperable patients as palliative [2]. These elements have the potential to reduce the recovery time of the patients and thus the costs for the hospitals.

Temperature monitoring is considered to be particularly beneficial for adjusting delivered energy settings during treatment. It has been shown that temperature can also be used as a clear end-point to achieve complete tumor ablation and to minimize recurrence [3]. Furthermore, the efficacy of hyperthermal treatment planning tools in therapy management can be strengthened by feedback in the form of accurately measured tissue temperatures [4]. During recent decades, several thermometric techniques have been proposed to guide ablation-based treatments in research, and more recently in clinical settings [5]. These methods can be divided into invasive (contact) and non-invasive (contactless) [6]. In the case of non-invasive thermometry, measurements of temperature change are performed in the absence of contact between the apparatus and the internal body, and inferred from images of temperature-dependent tissue properties; the best-known approaches are based on Magnetic Resonance (MR), Computed Tomography (CT), Ultrasound (US) imaging, and, recently, shear wave elastography [5,7,8,9,10]. Even though there exist clear advantages related to the absence of contact and the possibility of obtaining a 3D temperature map, image-based thermometry is not mature enough to be used as a clinical tool for the monitoring of all thermal procedures. In fact, (i) MR thermometry, which is considered the current clinical gold standard among non-invasive thermometry, needs ad hoc designed sequences, and its thermal sensitivity depends on the types of tissue unless a proton resonance frequency shift technique is used [9,11,12,13]. Moreover, the MR scanner can only be operated in conjunction with MR-compatible devices; (ii) CT-thermometry uses ionizing radiation (X-Ray), hence the first concern is related to the dose to the patient. Moreover, its thermal sensitivity is tissue-dependent, and there exist only preliminary studies regarding its in vivo feasibility assessment [14,15,16]; (iii) US-thermometry seems promising but only in a temperature range up to about 50 °C; moreover, the accuracy of this technique can be poor using particular methods (e.g., thermometry based on the changes of the speed of sound with temperature) when the temperature is close to 60 °C, and the thermal sensitivity depends on the nature of the tissue.

Invasive methods require the sensor to be inserted into the target tissue, but are much more cost-effective than imaging systems, and in some commercially available models, sensors are embedded within the energy-delivering probe (e.g., StarBurst^®^ XL RFA Device [17]), thus minimizing the invasiveness of the procedure.

Currently, the most frequently used sensors are thermocouples and fiber optic-based sensors (FOSs). Thermocouples, which consist of two metallic wires, are inexpensive, quite accurate (~1 °C), and have a relatively short response time (it strongly depends on the probe diameter, and can be much shorter than 1 s). On the other hand, substantial measurement error can occur for two main reasons: (i) the direct absorption of light by the metallic wires during LA, and the sonication during HIFU can result in substantial temperature overestimation [18,19]; (ii) the high heat conductivity of metallic wires can also cause temperature overestimation (for cryoablation) or underestimation (for hyperthermal treatment) [20]. Moreover, the metallic wires potentially cause significant image artifacts in CT- or MR-guided thermal procedures.

In particular configurations, fiber optic technology allows for overcoming these hurdles: thanks to their constitution (glass or polymer), FOSs are not prone to overestimation caused by light absorption, and have low heat conductivity (Silica Glass is an excellent thermal insulator). Moreover, MR-compatible FOSs can be used during CT- and MR-guided thermal procedures [21]. These features make the technology of FOSs particularly attractive for temperature monitoring during thermal treatments.

There exist several types of FOSs, which are underpinned by different working principles, and are usually divided in two classes [22]: (i) intrinsic, where the optical fiber constitutes the sensing element; and (ii) extrinsic, where the optical fiber is just a medium for conveying the light to and from a separate element or space. Among the large number of FOSs, only two kinds are extensively used for temperature measurements during thermal treatments, namely: Fiber Bragg Grating sensors (FBGs) and fluoroptic sensors. In addition to the listed valuable characteristics, FBGs are also able to perform distributed, quasi-distributed, and multi-point measurements, allowing the measurement of temperature in different points of the tissue by inserting a single small-sized element (e.g., an optical fiber with an outer diameter of hundreds of micrometers).

This article reviews the state of the art of FOSs (in particular, FBGs and fluoroptic sensors) used for temperature monitoring of thermal treatments. Throughout this paper a critical description of the main advantages and disadvantages of these two sensors is provided together with a consideration of the different thermal treatments. For the sake of clarity the article is arranged into two main parts: in the first part the essential physical principles of the most used thermal procedures are described, as well as the importance of temperature monitoring during these treatments; in the second part the measuring principles, advantages, and weaknesses of the FBGs and fluoroptic sensors are described, as well as their applications in the field of interest. Finally, emerging solutions based on fiber optic technology are proposed to improve temperature monitoring during thermal treatments.

## 2. Thermal Treatments and Temperature Monitoring

**Figure 1:**
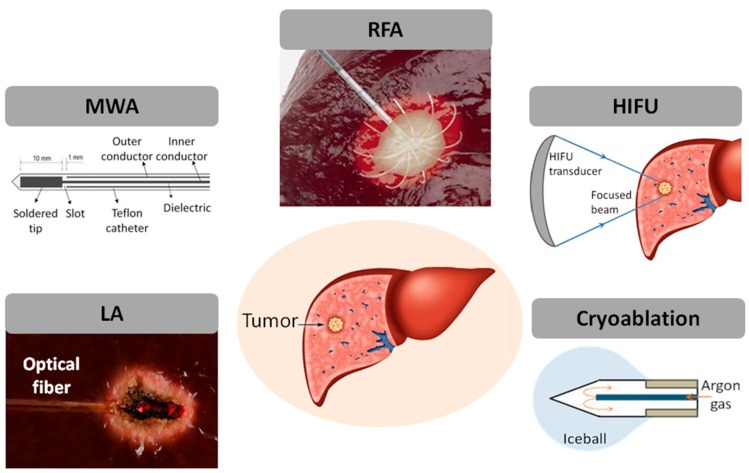
Minimally invasive thermal treatments for tumor removal: laser ablation (LA); microwave ablation (MWA); radiofrequency ablation (RFA); high intensity focused ultrasound (HIFU); and cryoablation.

This section focuses on the physics of the most used minimally invasive techniques and on their applications in tumor treatment (Figure 1). Moreover, the importance of temperature monitoring during these treatments and the requirements to be fulfilled by thermometry techniques employed in this field are described.

### 2.1. Thermal Treatment Modalities: Essential Physics and Applications

***Laser Ablation.*** Laser light is guided by a thin fiber-optic applicator (diameter of 300–600 μm), in contact with the tumor. The therapy is based on the photothermal effects related to the absorption of laser light by the biological tissue, which results in tissue hyperthermia around the applicator. The removal of the tumor can be attributed to several mechanisms, including plasma formation, tissue vaporization, combustion, and explosive tissue fragmentation. Typical light wavelengths used for cancer removal are 980 nm (diode laser) and 1064 nm (Nd:YAG laser), which guarantee optimal penetration depth of light into the tissue. The efficacy of the therapy is related to several parameters: laser settings (power, energy, time of exposition), laser wavelength, emission modalities of the applicator, as well as the absorption characteristics of the target tissue. LA is currently used for treatment of cancer of the liver, benign and malignant thyroid nodules, prostate and benign prostatic hyperplasia, and, more recently, cancer of the pancreas [23,24,25,26].

***Microwave Ablation.*** The interaction between polar water molecules and the electromagnetic field applied through an antenna forces dipoles to continuously realign with the electromagnetic field, thus producing frictional energy that is then converted into heat. Commercial MWA systems work in the range of 915 MHz–2.45 GHz, delivering power up to 100 W. They are relatively insensitive to tissue features (e.g., impedance, perfusion, etc.), making this technology more attractive than LA and RFA. Moreover, new devices achieve a more reproducible and controlled ablation area by controlling the change of dielectric properties during treatment, thanks to the integration of some levels of control (e.g., thermal control, field control, and wavelength control) [27]. MWA has been recently introduced for the treatment of lung and liver tumor, and some data are also available about pancreatic cancer [28,29].

***Radiofrequency Ablation.*** RFA requires an electrode that couples the RF current to the surrounding tissue. The electrode comprises a metal spindle, which is insulated with the exception of the exposed conductive tip, and a wide return electrode placed on the patient’s skin. The RF generator produces a voltage between the electrode tip and the grounding pad, establishing lines of electrical field within the patient’s body between the two electrodes. The typical RF range used for this treatment is <1 MHz, which allows for the oscillation of the electric field and, consequently, the oscillatory movements of tissue-based ions (current), with a velocity proportional to the electric field intensity. The mechanism of tissue heating with RFA is based on frictional (or resistive) energy loss associated with this current. A major strength of the RFA technique is the feasibility of adapting the geometry of the electrode to the shape of the tumor to be treated, as well as the use of multi-probes to increase the ablation area [30,31].

***High-Intensity Focused Ultrasound Ablation.*** An ultrasound wave produced by an oscillating piezoelectric crystal (frequencies ranging from 0.2 MHz to 3.5 MHz) in the generator outside the body is focused on the target region. HIFU transducers deliver ultrasound with intensities (power densities) in the range of 100–10000 W∙cm^−2^ to the focal region, with peak compression pressures up to 70 MPa, and peak rarefaction pressures up to 20 MPa (these values depend on the target tissue). The predominant mechanisms involved in the tissue damage are thermal (conversion of mechanical energy into heat) and purely mechanical. Concerning the latter, stable cavitation, inertial cavitation, and micro-streaming are responsible for the oscillation of the size of the bubble when exposed to a low-pressure acoustic field (stable cavitation) and violent oscillations of the bubble and its consecutive rapid growth during the rarefaction phase, when they reach their size of resonance (inertial cavitation). The oscillating motion of stable cavitation causes rapid movement of fluid near the bubble (micro-streaming) and induces cell apoptosis. Currently, HIFU is used for the ablation of tumors in the liver, prostate, breast, and kidney, and benign thyroid nodules [32,33,34].

Laser, microwave, radiofrequency, and HIFU ablation are hyperthermal procedures, since they rely on the increase of tissue temperature, and on its history, to produce an effective outcome. In fact, at temperatures of about 43 °C, irreversible cell damage starts to occur after prolonged exposure (from 30 to 60 min); when the temperature is above 60 °C, protein denaturation begins to occur, and the time required to achieve irre­versible damage decreases exponentially; usually, a temperature of about 80–100 °C allows for obtaining a fast ablation of the tissue, due to the water evaporation and the consequent damage to cell membranes [35].

***Cryoablation.*** This technique is performed percutaneously under imaging guidance and, differently from the previous ones, provides the therapeutic destruction of a tumor by freezing: ice formation within the extracellular space causes an osmotic gradient, responsible for tissue dehydration. At the iceball boundary the temperature is 0 °C, whereas the lethal values between −50 °C and −20 °C are achieved within 5 mm inside the iceball edge [36]. The procedure is performed by means of a cryoprobe and a cryogenic freezing unit: the unit allows a high-pressure gas (e.g., argon) to circulate within the lumen of the cryoprobe. The low pressure within the lumen causes rapid expansion of gas, which results in a temperature decrease and in the formation of an iceball around the probe tip. CT and MR images are particularly recommended for the monitoring of cryoablation [37].

### 2.2. Temperature Monitoring during Thermal Treatments: Importance and Requirements

**Figure 2:**
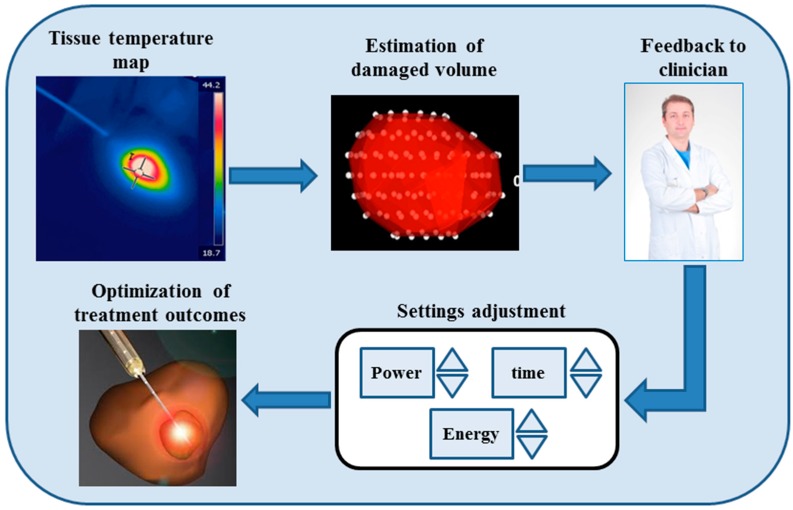
Concept of the utility related to the temperature monitoring.

The common goal of the thermal treatments is selective tumor removal without damaging healthy tissue. Therefore, it is important that the accurate localization of the tumor is achieved in conjunction with accurate identification of its features (i.e., geometry and contours) in order to perform an optimal placement of the applicator within the tumor (for RFA, LA, MWA, and Cryoablation) or to focus the ultrasound beams on the tumor (for HIFU). However, accurate tumor localization alone is not sufficient to obtain a selective treatment. The tumor damage depends on both temperature and exposure time, since both the exposure time and temperature contribute to cell death [14]. These two parameters were taken into account considering the thermal dose via the Arrhenius rate analysis [38]. As a consequence, temperature monitoring during the procedure facilitates more accurate assessment of the region affected by thermal damage, hence temperature feedback may be particularly beneficial for on-line adjustment of the treatment settings during the procedure and allows the operator to better visualize the running procedure and to be notified in real time about its end-point [3] (Figure 2).

It is important to focus on the system performance required to obtain an effective monitoring of the therapy, particularly in terms of spatial and temporal resolution as well as accuracy. They depend on the types of treatment: for instance, a short treatment such as HIFU requires better performance in terms of temporal resolution than LA, since it reaches the coagulative temperature within less than 30 s, as opposed to the longer treatment time of LA (i.e., usually 5–15 min). Nevertheless, measurement systems able to provide a temporal resolution of about 1 s can guarantee a more accurately controlled treatment in any therapeutic scenario [5]. Regarding spatial resolution, it is very important to consider the temperature gradient around the applicator: the sharper the thermal gradient, the better the spatial resolution achieved. A spatial resolution of 1 mm fulfils the stricter criteria but is difficult to obtain. Finally, an accuracy better than 1–2 °C is recommended [39].

Moreover, because of the high spatial gradient of the temperature, it is worth noting that the temperature change should be accurately controlled in proximity to the boundaries of the tumor, aiming to maintain the surrounding parenchyma at body temperature. The surrounding tissue is therefore preserved from unwanted damage [40]. In particular, in clinical practice, a 1-cm margin of apparently healthy tissue at the periphery of the tumor (known as a “safety zone”) is also thermally treated, to reduce the risk of incomplete ablation [41]. In any case the control of the temperature close to the applicator tip, especially during laser ablation, is crucial for the life of the applicator itself, since it could be damaged by the high temperature, resulting in its emitting properties being changed and hence affecting the efficacy of the treatment.

## 3. Fiber Optic Sensors for Temperature Monitoring during Thermal Treatments: Working Principles and Metrological Properties

Temperature sensors based on optical fibers have key advantages compared to their electrical counter-parts, such as micro electro-mechanical systems (MEMS), in terms of performance, size (of sensing area and cabling), and possibility of integration. Fluorescence-based thermometry was first commercialized in 1978 [42]; fluoroptic systems have been supporting thermal measurements in hyperthermia particularly in the last decade. Lately, the new developments in FBG sensors, and particularly the consolidation of drawing tower-based fabrication methods [43], have reduced costs and improved spatial resolution of FBG sensors to 0.5 to 2 sensors/cm, within the same fiber. Emerging techniques allow “hyper-dense” sensing, reducing the spatial resolution below a millimeter: two notable examples are chirped FBGs, which extend the FBG principle [44], and distributed sensing systems based on swept-wavelength interferometry for Rayleigh scattering analysis [45].

### 3.1. Fluorescence-Based Sensors

***Working Principle.*** Fluorescence-based sensors, incorporated in optical fibers, are based on the principle of operation of fluorescence lifetime measurement [42,46]. There was a considerable research effort on fluorescence-based thermometry during the 1990s, during which the principle of fluorescence decay of phosphor materials was implemented in optical fibers [47,48].

**Figure 3:**
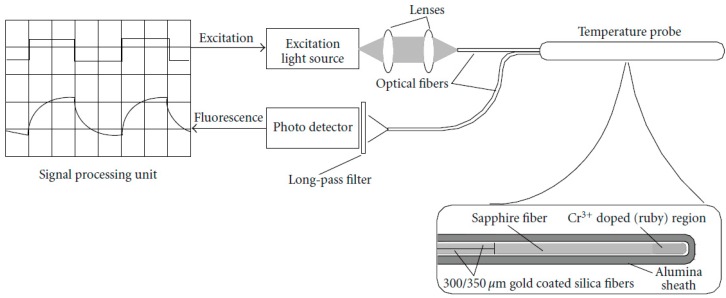
Schematic diagram of fiber optic sensor based on fluorescence lifetime measurement (from [50]).

Extrinsic fluoroscopy is based on the measurement of fluorescence decay time, induced in a fluorescent material such as ruby, alexandrite, thulium, or several rare-earth materials. Figure 3 shows a typical extrinsic fluorescence-based thermometric system, as proposed by Takahashi et al. [49]; the schematic is included in the review paper by Yu and Chow [50]. A light source, internally modulated with a square-wave pattern, and coupled inside a standard optical fiber is used to excite the phosphor; the probe is a Cr^3+^-doped region on the tip of a sapphire fiber, spliced to a silica fiber and encapsulated in an alumina sheath. A high-speed photodetector is used to record the decay time of the fluorescent material. Usually, the temperature value is extracted from the sensor output by the following two steps: (i) the sensing element is excited by a light pulse; (ii) after this stimulus, the fluorescent signal decays with an exponential pattern. The time constant of the exponential trend depends on the temperature, hence it can be considered an indirect measurement of temperature [46,47]. These probes have an approximate cost of about $100.

As the exponential decay is limited to a few μs, fluoroscopic sensors typically have fast responses.

***Technical and Metrological Features.*** In addition, most rare-earth materials are compatible with operation from room temperature to well over 200 °C, as well as operation below −40 °C. The system proposed by Wickersheim and Sun [51], and industrialized by Luxtron (now LumaSense, Inc, 3301 Leonard Court, Santa Clara, CA, USA [42]), is based on a fluorescent phosphor based on Eu^3+^-doped Gd_2_O_2_S) material; this system operates in the range of −100–290 °C with 0.1 °C accuracy. Detection speed, accuracy, and the possibility of using the fiber-probe as a disposable unit are attractive features for fluorescence-based systems, and for this reason several patents have been developed for incorporation of one or multiple fiber-optic temperature sensors in thermal ablation devices. Most notably, Vaguine [52] patented a system for microwave selective ablation supported by fluoroscope thermometers.

***Main Applications.*** The system proposed in [51], commonly known as a fluoroptic sensor, has been extensively used for the monitoring of tissue temperature in all thermal procedures (i.e., LA, MWA, RFA, HIFU, and cryoablation), from 1995 to date (see Table 1).

### 3.2. Fiber Bragg Grating (FBG)

***Working Principle.*** Fiber Bragg grating (FBG) sensors [53] are the most popular approach for modern fiber-optic sensing. An FBG is a wavelength-selective notch filter that reflects a narrow spectrum around a single peak wavelength; when temperature variations are applied to the FBG structure, the FBG spectrum shifts with near-perfect constant sensitivity. Hence, the wavelength that corresponds to the maximum value of the reflected spectrum intensity, called the Bragg wavelength (λ_B_), can be used to estimate the temperature. Since the FBG reflects a narrow spectrum and is transparent to all other wavelengths, it is possible to deploy an array of multiple FBGs fabricated on the same fiber, each having a different center wavelength, hence making use of wavelength division multiplexing (WDM). In this configuration, the FBG-based systems acquire a new dimension for biomedical sensing, as they allow several miniature sensors to be hosted on the same fiber, maximizing the sensing capacity. The cost of an FBG sensor is about $35 or less. However the system used to interrogate the sensor is more expensive (in the order of $10k).

**Figure 4:**
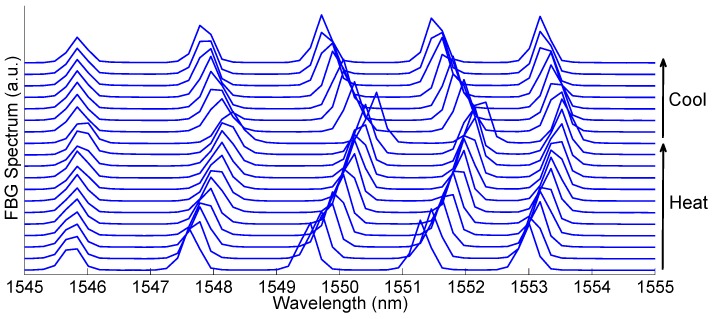
Application of FBG sensors to RF ablation [54,55]: the spectrum of an array of five FBGs is recorded during the heating and cooling stages; spectra on the chart after every 20 s of application.

***Technical and Meteorological Features.*** In the range of measurement required for thermal ablation (i.e., 30–100 °C), FBGs have constant sensitivity, whose typical value is ~10 pm·°C^−1^. Figure 4 shows five FBG sensors inscribed on the same fiber, applied for RF ablation [54,55]; each FBG has 0.5 cm active length, with 1 FBG/cm sensing capacity, and the distance between each peak wavelength is 1.8 nm; this result corresponds to one of the most recent examples of FBG sensing in thermal ablation. The response of the five FBG arrays during heating and cooling is shown in Figure 4. Compared to fluorescence-based sensors, the possibility of performing WDM and therefore incorporating several sensors in a single fiber, with narrow density, is a key advantage of FBG sensors. By detecting the FBG spectrum with an interrogator, and applying post-processing, it is possible to retrieve the temperature for each sensor with 0.1 °C accuracy.

The technology behind the FBG sensors is progressing rapidly with many recent advances in fabrication techniques. Most notably, the consolidation of the drawing tower fabrication of FBG arrays, established in the so-called draw-tower gratings (DTGs) [56] that have been industrialized by FBGS International [57], is providing significant metrological meteorological advantages over more traditional FBG fabrication techniques, based on the exposure of a fiber to UV light through a phase mask. DTGs can be fabricated with precise positioning: there is a one-to-one correspondence to the Bragg wavelength of each sensor composing the array, and its geometrical location along the fiber; this is essential in hyperthermia to provide a reliable temperature pattern reconstruction. Mechanical strength is also increased as the DTG fabrication process does not require stripping and recoating the fiber buffer, maintaining the original robustness and thickness. In addition, DTGs are usually fabricated on ormoceramic bend-insensitive fibers. Currently, commercial DTG arrays achieve 1 FBG/cm density on a single fiber.

More recently, Geernaert et al. [58] set up at Cyprus University of Technology a novel technique for FBG fabrication, which employs point-by-point inscription using a femtosecond laser. This technique has the potential to improve the sensing capacity, as it may allow in the near future the fabrication of highly reflective FBGs with <1 mm length, packed in a dense array.

***Main Applications.*** FBGs have been largely used for the monitoring of tissue temperature during LA, RFA, and more recently during MWA and cryoablation. To date, no uses during HIFU are reported (see Table 1).

### 3.3. Chirped FBG

***Working Principle.*** A chirped FBG behaves as a continuous chain of FBGs, each having a different peak wavelength. The most interesting configuration is the linearly chirped FBG (LCFBG), in which the Bragg wavelength varies linearly in the space [44].

***Technical and*** Metrological ***Meteorological Features.*** Chirped FBGs are fabricated for lengths of 1.5 cm up to 5 cm, and have a bandwidth ranging between 5 nm to 50 nm. From a metrological meteorological perspective, LCFBG behaves as a chain of sensors; its spectrum results from the entire temperature pattern, across all sensors [53]. The use of LCFBG in spatially resolved temperature measurement is still at a relatively early stage, and the first application in hyperthermia was in 2014 [45]. By using a LCFBG in lieu of standard FBG arrays, the spatial resolution drops well below 1 mm, and is mainly limited by the capability of the decoding system to resolve the temperature pattern from the LCFBG spectrum. The typical cost of a chirped FBG is about $200–300. The back-reflected spectrum from the chirped FBG can be recorded by the same interrogator used for uniform FBG (costs about $10k), but custom software can be developed to decode the signal and to estimate temperature, since no commercially available software currently exists.

***Main Applications.*** Chirped FBGs are gaining popularity in the field of tissue temperature monitoring during thermal procedures (in particular RFA) only recently (see Table 1). Preliminary results reported by Tosi et al. show a spatial resolution of 75 μm on 1.5 cm length. The decoding technique is, however, effective, mainly for monotonic temperature patterns. Current research is aimed at developing fast decoding algorithms for non-monotonic temperature patterns, as typically obtained in thermal ablation.

### 3.4. Rayleigh Scattering Distributed Sensing

***Working Principle.*** Distributed temperature sensing (DTS) utilizes a different approach from the previous technologies, as it makes use of a standard fiber as sensor; decoding is performed in the time or frequency domain, by measuring the Rayleigh backscattering pattern [58,59,60]. Currently, the gold standard instrument of DTS for dense spatially resolved thermal measurement is the Luna OBR4600 [61], which is based on the principle of operation of swept-wavelength interferometry developed by Gifford et al. [59]. Such a DTS system is capable of recording the Rayleigh backscattering signature, originating in the sensing fiber, and resolving it with sub-mm spatial accuracy. These sensors are developed using standard single mode fibers (the cost is negligible), but they need an expensive interrogator to analyze and record the signal ($50–120k).

***Technical and*** metrological ***Meteorological Features.*** Performance depends on a tight trade-off between spatial resolution, accuracy, active length, and sampling time. This system was employed for the first time in hyperthermia by Macchi et al. in 2014 [45], achieving 200 μm spatial resolution and approximately 0.5 °C accuracy, for 1 Hz measurement rate. As the system operates in a standard fiber, without the need to fabricate any structure, it allows the development of a low-cost disposable probe; on the other hand, the interrogator cost is at least one order of magnitude higher than the other fiber-optic sensing system and the ablation devices.

***Main Applications.*** To the best of our knowledge, only one study [45] have employed Rayleigh scattering distributed sensing systems in medical scenarios, even though they are a promising solution for the measurement of distributed temperature or a thermal gradient.

## 4. Application of FOSs in Temperature Monitoring during Thermal Treatment

Commercial systems based on FOS were introduced for monitoring hyperthermal effects in the 1980s; later Vaguine and colleagues proposed a multi-probe optical sensors for MWA monitoring [52]. In the late 1990s, Rao et al. fostered the use of FBGs for temperature monitoring during hyperthermia. They developed a novel system enclosing an FBG sensor array in a protective sleeve (diameter of 0.5 mm) to avoid measurement errors due to strain [62]. The same group later tested an upgraded version of the system inside an MR scanner with a magnetic field of 4.7 T. The probe revealed a temperature resolution of 0.2 °C and an accuracy of 0.8 °C, for temperatures ranging from 25 °C to 60 °C [63]. The first in vivo trial with this novel probe was carried out in 2000 by Webb et al. on diseased livers and healthy kidneys of rabbits undergoing hyperthermia [64]. Although in this work the temperature increment was caused by heating a metal probe, it paved the way for the numerous studies focusing on the use of FOSs during in vivo trials. The following subsections provide a detailed review of the most significant works focusing on the use of FOSs for temperature monitoring during thermal treatments.

### 4.1. Applications of FOSs during Laser Ablation

Several thermometric techniques used for the monitoring of tissue temperature during LA have been presented by Saccomandi et al., in their recent review [6]. The intrinsic characteristic of LA to be performed inside an MR environment, because of the use of MRI-compatible instrumentation (i.e., optical fibers guiding laser beams), promoted the use of FOSs for temperature monitoring.

Fluoroptic sensors have been employed largely to provide the reference temperature in the calibration of MRI-based thermometry, on both ex vivo organs and tissue equivalent phantoms. Nevertheless, efforts have been made to characterize the performance of fluoroptic sensors in thermometry during laser irradiation because of the presence of a measurement error caused by the self-heating of fluoroptic sensors [65]. This artifact mainly depends on the black pigments in the coating of the fluoroptic probe. Reid et al. found that measurement error induced by self-heating of the fluoroptic probe in presence of laser irradiation cannot be neglected if the distance between the laser applicator and the sensor is less than 4 mm [66].

One of the first applications of FBG for thermometry in LA was presented by Ding et al. in 2010, who developed a distributed FBG sensor with a length of 10 mm, encapsulated within a glass capillary, and used it to monitor temperature distribution in an ex vivo liver and in an in vivo mouse [40,67]. The authors assumed that a uniform grating turns into a chirped grating in a non-uniform temperature field. The algorithm implemented was useful to dynamically control the temperature of the target at 43 °C, and the temperature at the edge and outside the target at 38 °C.

Several studies have been carried out from 2012 to date by the group of Saccomandi and Schena, aiming to measure the temperature distribution in a pancreas undergoing LA, at different laser settings. They used non-encapsulated single-point FBGs of 10 mm length, and determined that FBGs do not experience any appreciable mechanical strain because of the relaxation of ex vivo tissue under its weight (error less than 1 °C) [68,69]. The same research group evaluated the influence of sensor length in the presence of a high thermal gradient: by comparing the output of 1 mm-long FBG with the response of 10 mm-long FBG; they demonstrated that small FBGs are recommended for accurate temperature measurement close to the laser applicator [70].

An issue regarding the use of FBG for in vivo temperature monitoring is the presence of measurement errors due to patient respiratory movements, hence solutions for the encapsulation of FBGs with a protective sleeve have been proposed. Two surgical needles were used to encapsulate a 10 mm long FBG with two materials, i.e., epoxy adhesive and thermal paste. The needle-like probes showed different sensitivity (0.01 nm·°C^−1^ for FBG with thermal paste vs. 0.027 nm∙°C^−1^ for FBG with epoxy adhesive), a similar time constant (~100 ms), and the artifact due to self-heating of the metal has been evaluated and corrected. Aiming to assess the measurement error due to strain on the probes caused by the breathing movement of the patient, the displacement of ex vivo liver has been simulated. The encapsulation proved its efficacy, while showing an error less than 0.5 °C vs. 2.3 °C experienced by non-encapsulated FBG [71]. Similar needle-like probes have been developed by using commercially available MRI-compatible needles, tested on an ex vivo liver undergoing LA and MWA, and characterized in MR 1.5 T scanner [72].

In further studies, multipoint non-encapsulated FBG sensors have been employed to provide the reference temperature in both CT and MRI thermometry calibration [73,74].

Recently, Liu et al. developed a laser applicator integrating FBG sensors for temperature measurement during LA. A double cladding fiber, i.e., a type of fiber commonly employed in the realization of high-power fiber lasers, has been exploited to combine laser beam delivery and sensing capability in the same fiber. The FBG showed response time of 50 ms. The probe has also been successfully tested on a tissue phantom, but further experiments on ex vivo organs will encourage its use in clinical practice [75].

### 4.2. Applications of FOSs during Microwave Ablation

Fluoroptic sensors have been largely used during MWA as reference devices for MRI-based thermometry, and to validate theoretical models describing thermal effects on tissue undergoing the treatment.

In 2006, Demura et al. used fluoroptic sensors to calibrate spatially resolved MRI thermometry for real-time monitoring of phantoms and ex vivo animal organs to realize a temperature map during MWA performed with MR compatible antenna [76].

In 2007, Yang measured ex vivo bovine liver temperature during MWA using fluoroptic sensors, with the aim of analyzing the effects of water vaporization during hyperthermia [77]. In 2011, Rubio et al. used four fluoroptic sensors to measure the temperature distribution of ex vivo swine muscle undergoing MWA (2.45 GHz, 10 W, 3 min), aiming to validate a predictive model based on FEM simulation [78]. Chen et al. [79] used these sensors in clinical settings on five patients diagnosed with prostate cancer. The MWA system was MRI-compatible, in order to perform MR-thermometry, and two fluoroptic sensors were inserted into the patients for the simultaneous measurement of temperature in two different locations.

The use of FBG for temperature monitoring during MWA has been a relatively recent development. In 2010 Saxena et al. [80] proposed a polymer-coated FBG embedding 10 gratings into a 125 μm diameter glass optical fiber. The fiber was embedded in a 0.5 mm diameter probe, and the 10 FBGs were equally spaced at 5 mm intervals. The grating spectra were separated by 3.5 nm, aiming to avoid the wavelength overlap caused by temperature change. The polymeric coating allows achieving high temperature sensitivity: its coefficient of thermal expansion being higher than fused silica fiber amplifies the thermally induced stresses on the gratings: sensitivity of 23 ± 7 pm∙°C^−1^ in the range 25–60 °C, vs. the sensitivity of uncoated FBG of 8.6 ± 0.2 pm∙°C^−1^. The larger sensitivity uncertainty is due to the variability of polymer thickness manually deposited on the fiber. The sensor has been tested on a muscle equivalent phantom undergoing MWA, and compared with the response of commercially available thermometers.

Recently, Saccomandi et al. have compared the performances of two MWA systems (915 MHz and 2.45 GHz frequencies) in terms of the temperature map and its reproducibility. They used FBG sensors of 10 mm length, embedded in needles (described in [72]) placed at several distances from the antenna (from 5 mm to 30 mm) [81].

Also, other types of FOSs have been used for temperature monitoring during MWA. Pennisi et al. designed a new prototyped sensor and tested it on a phantom undergoing MWA. The sensor’s working principle is based on the thermal dependence of refractive index of the material surrounding the cladding, entailing change of power light transmitted through the fiber [82]. The optical power transmitted by the fiber increases with temperature in a non-linear manner in the range 18–50 °C. The non-linear trend is due to the change in refractive index of oil filling the glass tube with respect to the stable refractive index of the cladding: when the refractive index of the oil equals the index of the cladding, the transmitted power reaches the maximum value, and beyond this a saturation effect occurs.

The sensor was fabricated by using a multimode and commercial available patch-cord. A 2 cm jacket length, buffer, and part of the glass cladding are removed from a long length of fiber, afterwards encapsulated within a 1.1 mm diameter glass tube filled with oil. Finally, both ends of the tube are sealed with epoxy resin. The time constant of the sensor was reported as 1.9 s.

In 2011, Ji and Brace used a commercial fiber optic temperature sensor (Neoptix T1 probe, Ville de Québec, QC, Canada) during ex vivo liver MWA, based on a well-known and reproducible semiconductor phenomenon: the band-gap variation in the absorption spectrum of semiconductor crystal gallium arsenide (GaAs) with temperature. As the temperature of GaAs increases, its transmission spectrum shifts to higher wavelengths [83].

### 4.3. Applications of FOSs during Radiofrequency Ablation

Temperature distribution during RFA is the most widely studied parameter due to the extensive use of the RFA technique for tumor treatment. For instance, fluoroptic sensors have been employed in an in vivo canine model to investigate the effects of RFA with active electrode cooling, and on phantoms to assess the change of electrical conductivity with temperature and the temperature distribution around different models of antennae [44,84,85,86,87,88]. Other studies employed fluoroptic sensors as a reference for MRI thermometry on ex vivo models and on patients undergoing RFA [89,90].

Studies focused on the design and prototyping of FOSs for temperature monitoring during RFA have been carried out by Tosi et al. [55]. In 2014 they developed a multi-point FBG probe, embedding five gratings of 5 mm length that were equally spaced at 10 mm intervals. The FBG array has a uniform spectral spacing (1.8 nm between each central Bragg wavelength, 1546.0–1553.2 nm), and the thermo-optic FBG coefficient is 11.66 pm·°C^−1^. The array was mounted on an RF needle and tested during thermal procedures on an ex vivo porcine liver. The same group investigated the use of chirped FBG, suitable for temperature gradient measurement, with the following characteristics: length of 15 mm, linear chirp parameter of 2.22 nm/mm, and a thermo-optic coefficient of 10.2 pm·°C^−1^ exhibited by the FBG in uniform heating conditions. The sensor has been calibrated in the range 22–95 °C, and an ad hoc algorithm has been proposed to correlate the change of temperature spatial gradient with the reflected spectrum, before testing the transducer on the ex vivo liver undergoing RFA [88].

A further interesting study focused on FOSs for simultaneous temperature and pressure measurements. The device combines the two FBG-based solutions (FBG array and chirped FBG), with a Fabry–Pèrot interferometer for temperature and pressure measurement, respectively [54]. The device was tested on an ex vivo porcine liver, measuring temperature values up to 160 °C at the end of the RFA application period.

### 4.4. Temperature Monitoring during HIFU Ablation by FOSs

Most studies are limited to a description of the use of commercial FOS thermometers for HIFU temperature monitoring or to provide a reference for image-based thermometry. For instance, one of the first thermometric studies using a fluoroptic sensor on tissue undergoing HIFU was described by Bohris et al. for the calibration of MRI-based thermometry on porcine muscle and fatty tissue [89]. In 1997 Jenne et al. presented the use of CT-based thermometry on ex vivo swine muscle, and employed fluoroptic sensors as reference sensors [90]. Wong et al. subsequently used fluoroptic sensors on an oil-based phantom simulating the acoustic properties of a liver undergoing HIFU [91]; Ranjan et al. tested HIFU in vivo on rabbit Vx2 tumor models and measured the temperature by means of fluoroptic sensors and a conventional Neoptix T1 probe, during MRI-based thermometry [92]; Petrusca et al. employed four fluoroptic sensors on ex vivo turkey tissue undergoing HIFU, as a reference device for MRI-based thermometry [93].

A different method was used by Morris et al. [94], who performed a simultaneous measurement of temperature and acoustic pressure in samples undergoing HIFU using a Fabry-Pérot fiber-optic ultrasonic hydrophone. The transduction mechanism is based on the detection of acoustically- and thermally-induced changes of thickness in a polymer film Fabry-Pérot interferometer deposited at the tip of a single mode optical fiber. The sensor was tested in the temperature range 25–80 °C, and showed a linear response up to 70 °C, with a resolution of 0.34 °C using an oil-gelatin phantom undergoing HIFU.

Other FOSs based on FBGs and Fabry-Pérot interferometers have also been employed in this application, but for the measurement of HIFU field [95,96,97]. For instance, an all-silica Fabry-Pérot FOS has been developed by Wang et al., with a very low wavelength change with temperature (0.000858 nm·°C^−1^), and good signal to noise ratio (42.8 dB) [96].

### 4.5. Temperature Monitoring during Cryoablation by FOSs

Limited studies have been undertaken on the monitoring of temperature distribution during cryoablation due to its recent introduction in the field of tumor treatment.

Fluoroptic sensors were initially employed in 2007 by Bouley et al., who investigated cryoablation outcomes on the in vivo dog prostate, and compared the effect with thermal damage induced by HIFU [36]. Recently, Favazza et al. employed temperature data provided by fluoroptic sensors to assess the impact of a urethral warmer on temperature distributions around cryoneedles during cryoablation, in a tissue mimicking phantom [98].

FBGs have been included in numerous studies involving temperature monitoring during cryoablation. In 2001, the research team of Samset started working on the development of FBG sensor for temperature monitoring in tissue undergoing cryoablation, and afterwards used this sensor to calibrate MR thermometry [99]. The distributed sensor was an optical fiber (cladding diameter 125 µm) embedding 10 FBGs. The center-to-center separation between the sensing elements was 6.5 mm and thus the total length of the sensor array was 58.5 mm. Two arrays were fabricated and mounted inside polyimide and titanium tubes, both materials having magnetic susceptibility close to that of tissue, with a total outer diameter of 1.4 mm. The sensor was calibrated in the range –189.5–100 °C. Mechanical stability and MRI compatibility were acceptable allowing routine use. The measurement system was tested on an in vivo porcine liver [100].

Gowardhan and Green used several multipoint sensors for temperature monitoring in 20 patients undergoing hypothermal treatment of the prostate (TMS, Multitemp^TM^ 1601, InvivoSense, Trondheim, Norway). This clinical study highlighted the importance of using a single sensor housing a number of sensors (in this study eight), rather than individual point sensors, as it provides the clinician with more simultaneous information about treatment, with minimal invasiveness [101].

The main characteristics of FOSs and their applications in LA, MWA, RFA, HIFU, and cryoablation are summarized in Table 1.

## 5. Discussion

The introduction of FOSs for monitoring the effects of thermal treatments can be considered a highly useful tool that will improve the outcomes of these treatments. Indeed, thermometry is one of the most popular approaches for controlling the amount of damage being imparted to tissue during the procedure, and hence it is gaining greater importance.

Image-based techniques (MR, CT, US, and shear wave thermometry) have two main advantages: they are contactless and they provide a three-dimensional temperature map of the target organ. Generally speaking, such methods are still under development. Even though several patents have been produced in the last decades, only MR thermometry has led to the release of a commercial tool (patented by Siemens^®^ [102]), but its use is still limited because of the limited availability of MR-compatible tools to perform the procedures. Moreover, their limited use is still related to the issue of dose (for CT-thermometry), the high costs of which constrain the availability of good imaging systems in some hospitals, and the ongoing research on the US and elastography techniques.

All these concerns foster the necessity of simple and safe measurement systems based on reliable sensors. For instance, sensor-based approaches are popular for in vivo temperature measurement. Among others, thermocouples, thermistors, and FOSs have been extensively employed for the monitoring of tissue temperature surrounding the applicator [6,19,40,44,45,52,54,55,64,67,71,72,75,89,100]; moreover, some commercial thermal systems have embedded one of these sensors in the applicator (e.g., thermocouples in the RF system in [17]). FOSs have many advantages including excellent metrological characteristics, electrical immunity, and safety, resulting in their compatibility for CT-guided and MR-guided procedures. These valuable characteristics motivate the use of these sensors in research and clinical practice and as reference sensors for other thermometric techniques during thermal treatments. In particular, FBGs have enormous application potential in monitoring of thermal treatment effects due to their ability to simultaneously measure temperature at several points within tissue by inserting a single fiber optic, as well as their small size and flexibility, allowing the possibility of reaching deep tissue. Moreover, in the field of thermal treatments the need for a fiber link between the grating at the point of measurement (the organ) and the distal detector (e.g., optical spectrum analyzer) is not an obstacle to the deployment of this sensor system as the grating or multiple gratings are formed within the fiber itself. A significant concern may be related to the cross-sensitivity to other parameters, such as strain, which can be caused by respiration or other types of motion of the patients (motion artifact). There are methods for compensating for or overcoming motion artifacts e.g., bend-insensitive fibers or embedding the FBGs in a metallic needle. If used wisely, they can allow the monitoring of multiple physiological parameters at the same time, e.g., pressure [103]. Additionally, the future advancement of the technology and the development of cheaper interrogator systems will further encourage the widespread use of FBGs.

## 6. Conclusions

In parallel with consolidated measurement techniques, the innovative approaches making use of chirped FBG and DTS systems may provide a significant breakthrough in fiber-optic thermometry, as they may make up for the main limitations in terms of spatial resolution, providing “hyper-dense” sensing with sub-mm spatial resolution. Current research is focused on improving the reliability and cost-effectiveness of such techniques in order to make them compliant with thermal ablation. Early-stage work during 2014–2015 has shown significant promise for innovative sensing tools.

Current applications of FOSs for temperature monitoring in the field of thermal treatment of tumors have been reviewed. Several advantages of FBGs with respect to electrical sensors have been highlighted, predicting a rapid future growth in the use of optical fiber-based sensors in monitoring thermal treatment and in general medicine. A crucial challenge for researchers in the field of FOSs is to increase clinician awareness about FBGs’ ability to overcome several concerns related to the use of traditional electrical sensors and to allow the development of more effective solutions and improved efficacy for thermal ablation treatment.

## Figures and Tables

**Table 1:** Performance and medical applications of FOSs used for temperature monitoring during thermal treatments.

First Author, Year, Ref	Kind of FOS	Thermal Treatment	Model (in vivo, ex vivo)	Kind of Sensor, Number, Size, Embedding	Features (Accuracy, Errors, Measurement Range, Constant Time, Frequency Response, Sensitivity)
Davidson et al., 2005 [65]	Fluoroptic sensors	LA	air, water	4 sensors	
Reid et al., 2001 [66]	Fluoroptic sensors	LA	air, water, agar–albumen phantom		
Yang et al., 2007 [77]	Fluoroptic sensors	MWA	ex vivo bovine liver	4 sensors inserted through biopsy needles	8–120 °C
Rubio et al., 2011 [78]	Fluoroptic sensors	MWA	ex vivo swine muscle	4 sensors	19–60 °C
Chen et al., 2000 [79]	Fluoroptic sensors	MWA	in vivo patients (prostate cancer)	2 sensors	measurement range: 37–70 °C
Nakagawa et al., 1998 [84], 2008 [88]	Fluoroptic sensors	RFA	In vivo canine model	4 sensors	
Solazzo et al., 2005 [85]	Fluoroptic sensors	RFA	Agar phantom		
Lobik et al., 2005 [87]	Fluoroptic sensors	RFA	Egg phantom		
van den Bosch et al., 2008 [12]	Fluoroptic sensors	RFA	3 women affected with breast cancer	4 sensors	
Viallon et al., 2010 [11]	Fluoroptic sensors	RFA	ex vivo tissue		
Bohris et al., 1995 [89]	Fluoroptic sensors	HIFU	ex vivo porcine muscle and fat		
Jenne et al., 1997 [90]	Fluoroptic sensors	HIFU	ex vivo porcine muscle		
Wong et al., 2007 [91]	Fluoroptic sensors	HIFU	oil phantom		
Ranjan et al., 2012 [92]	Fluoroptic sensors	HIFU	in vivo rabbit Vx2 tumor models	Neoptix T1 probe, Fluoroptic sensors	
Petrusca et al., 2015 [93]	Fluoroptic sensors	HIFU	ex vivo turkey tissue	Fluoroptic sensors	
Bouley et al., 2007 [36]	Fluoroptic sensors	Cryo	in vivo dog prostate	4 sensors	
Favazza et al., 2014 [98]	Fluoroptic sensors	Cryo	prostate mimicking phantom	4 sensors	
Saccomandi et al., 2012–2014 [68,69,70]	FBG	LA	ex vivo porcine pancreas	6 FBGs, 1 mm and 10 mm of length, non-encapsulated	
Polito et al., 2015 [71]	FBG	LA	ex vivo porcine liver	FBG 10 mm of length, encapsulated in metallic needle	measurement range: 20–80 °C sensitivity: from 0.01 nm∙°C^−1^ to 0.027 nm∙°C^−1^ time constant: 100 ms
Cappelli et al., 2015 [72]	FBG	LA	ex vivo porcine liver	3 FBGs 1mm of length, encapsulated in MRI compatible needle	measurement range: 20–80 °C sensitivity: 0.01 nm∙°C^−1^ time constant: 100 ms
Schena et al., 2013–2015 [73,74]	FBG	LA	ex vivo porcine liver and pancreas	4 FBGs 1mm of length, non-encapsulated	
Liu et al., 2015 [75]	FBG	LA	phantom	laser fiber integrating 2 FBGs	time constant: 100 ms
Saxena et al., 2010 [80]	FBG	MWA	muscle equivalent phantom	10 FBGs at distance of 5mm on fiber with 0.125 mm of diameter; coated with polymer (diameter of 0.5 mm)	measurement range: 20 °C–60 °C sensitivity: 23 ± 7 pm·°C^−1^ Accuracy: 0.25 °C stability over 10 h: 0.5 °C time constant: 2 s
Saccomandi et al., 2015 [81]	FBG	MWA	ex vivo porcine liver	FBG sensors (1cm of length)	
Tosi et al., 2014 [54]	FBG	RFA	ex vivo porcine liver	5 FBGs, 5 mm of length mounted on RF needle	FBG spectral spacing: 1.8 nm; Thermo-optic FBG coefficient: 11.66 pm·°C^−1^.
Tosi et al., 2014 [44]	FBG	RFA	ex vivo porcine liver	linearly chirped FBG 15 mm of length	Measurement range: 22–95 °C; Linear chirp parameter: 2.22 nm/mm; Thermo-optic coefficient: 10.2 pm·°C^−1^.
Tosi et al., 2015 [55]	FBG	RFA	ex vivo porcine liver	FBG array, linearly chirped FBG, Fabry–Pérot interferometer for pressure and temperature	see previous studies
Samset et al., 2001 [99,100]	FBG	Cryo	in vivo porcine liver	10 FBGs; 58.5 mm of length, outer diameter of 1.4mm	measurement range: −185–100 °C
Pennisi et al., 2002 [82]	change of refractive index	MWA	phantom	1 sensor based on change of refractive index of medium surrounding cladding; 20 mm of length	measurement range: 18–50 °C
Ji et al., 2011 [83]	band gap GaAs sensors	MWA	ex vivo bovine liver	4 band gap GaAs sensors; 0.4 mm GaAs sensitive area; probe material PTFE	measurement range: 20–130 °C Accuracy: 1 °C time constant: 250 ms
Macchi et al., 2014 [45]	DTS system	RFA	ex vivo porcine liver	DTS system based on swept laser interferometry	Spatial resolution: 0.2 mm; accuracy 0.5 °C; active region: 8 fiber spans, 3.6 cm each.
Morris et al., 2009 [95]	Fabry–Pérot interferometer	HIFU	oil-gelatin phantom	Fabry–Pérot interferometer	Measurement range: 25–80 °C (linear up to 70 °C); Resolution: 0.34 °C; rate of measurable temperature change: 67 °C·s^−1^.
