# Correction: Schena, E.; et al. Fiber Optic Sensors for Temperature Monitoring during Thermal Treatments: an Overview. *Sensors* 2016, *16*, 1144

**DOI:** 10.3390/s18041226

**Published:** 2018-04-17

**Authors:** Emiliano Schena, Daniele Tosi, Paola Saccomandi, Elfed Lewis, Taesung Kim

**Affiliations:** 1 Universita’ Campus Bio-Medico di Roma, Unit of Measurements and Biomedical Instrumentation, via Alvaro del Portillo 21, 00128 Roma, Italy; e.schena@unicampus.it; 2 School of Engineering, Nazarbayev University, 53 Kabanbay Batyr, 01000 Astana, Kazakhstan; daniele.tosi@nu.edu.kz; 3 Institute of Image-Guided Surgery (IHU), S/c Ircad, 1 place de l'Hôpital, 67091 Strasbourg Cedex, France; 4 Optical Fibre Sensors Research Centre (OFSRC), University of Limerick, V94 T9PX Limerick, Ireland; elfed.lewis@ul.ie; 5 School of Mechanical Engineering & SAINT, Sungkyunkwan University, 53 Myeongnyun-dong 3-ga, Jongno-gu, 110-745 Suwon, Korea; tkim@skku.edu

The author wishes to make the following correction to this paper [1], and to replace Figure 2: 
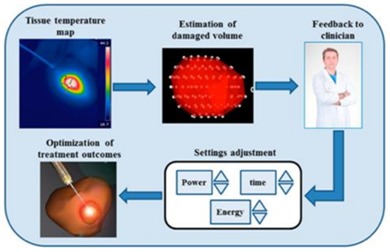

with: 
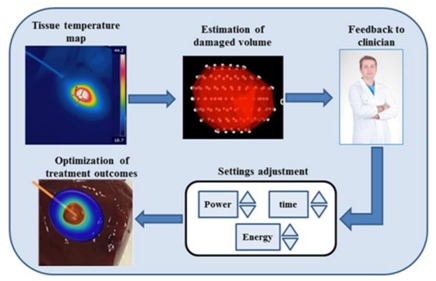


The authors would like to apologize for any inconvenience caused to the readers by these changes.
